# Helical Polariton Lasing from Topological Valleys in an Organic Crystalline Microcavity

**DOI:** 10.1002/advs.202203588

**Published:** 2022-08-21

**Authors:** Teng Long, Xuekai Ma, Jiahuan Ren, Feng Li, Qing Liao, Stefan Schumacher, Guillaume Malpuech, Dmitry Solnyshkov, Hongbing Fu

**Affiliations:** ^1^ Beijing Key Laboratory for Optical Materials and Photonic Devices Department of Chemistry Capital Normal University Beijing 100048 P. R. China; ^2^ Department of Physics and Center for Optoelectronics and Photonics Paderborn (CeOPP) Universität Paderborn Warburger Strasse 100 33098 Paderborn Germany; ^3^ Tianjin Key Laboratory of Molecular Optoelectronic Science School of Chemical Engineering and Technology Collaborative Innovation Center of Chemical Science and Engineering (Tianjin) Tianjin University Tianjin 300072 P. R. China; ^4^ Key Laboratory for Physical Electronics and Devices of the Ministry of Education & Shaanxi Key Lab of Information Photonic Technique School of Electronic Science and Engineering Faculty of Electronic and Information Engineering Xi'an Jiaotong University Xi'an 710049 China; ^5^ Wyant College of Optical Sciences University of Arizona Tucson AZ 85721 United States; ^6^ Institut Pascal PHOTON‐N2 Université Clermont Auvergne CNRS Clermont INP Clermont‐Ferrand F‐63000 France; ^7^ Institut Universitaire de France (IUF) Paris 75231 France

**Keywords:** helical polariton lasing, organic microcrystalline cavity, topological photonics, valley state

## Abstract

Topological photonics provides an important platform for the development of photonic devices with robust disorder‐immune light transport and controllable helicity. Mixing photons with excitons (or polaritons) gives rise to nontrivial polaritonic bands with chiral modes, allowing the manipulation of helical lasers in strongly coupled light‐matter systems. In this work, helical polariton lasing from topological valleys of an organic anisotropic microcrystalline cavity based on tailored local nontrivial band geometry is demonstrated. This polariton laser emits light of different helicity along different angular directions. The significantly enhanced chiral characteristics are achieved by the nonlinear relaxation process. Helical topological polariton lasers may provide a perfect platform for the exploration of novel topological phenomena that involve light‐matter interaction and the development of polariton‐based spintronic devices.

## Graphical abstract

Helical polariton lasers from topological valleys of an organic anisotropic microcrystalline cavity based on tailored local nontrivial band geometry is demonstrated. Helical topological polariton laser may provide a perfect platform for the exploration of novel topological phenomena that involve light‐matter interaction and the development of polariton‐based spintronic devices.

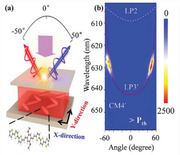

## 1 Introduction

The concepts of topology and of topological invariants have been introduced to describe the properties of physical systems a few decades ago. One spectacular consequence is that the topological character of gapped Bloch bands guarantees the existence of edge modes that can be unidirectional, offering the possibility of transport of electrons or photons immune against perturbations such as defects and disorder.^[^ ^1^, ^2^ ^]^ Another important consequence is the link between the Berry curvature and the angular momentum,^[^ ^3^ ^]^ which can manifest itself as spin‐valley locking,^[^ ^4^ ^]^ most well‐known in transitional metal dichalcogenides.^[^ ^5^ ^]^

Topological effects have been demonstrated in a range of physical systems, such as two‐dimensional (2D) electron gases,^[^ ^6^, ^7^ ^]^ cold atoms,^[^ ^8^ ^]^ mechanics,^[^ ^9^ ^]^ microwaves,^[^ ^10^ ^]^ and optical systems.^[^ ^11^, ^12^ ^]^ Topological photonics^[^ ^13^, ^14^ ^]^ was born in 2008 when an analog of the quantum anomalous Hall effect has been predicted^[^ ^15^ ^]^ and realized^[^ ^10^ ^]^ in the microwave range using a photonic crystal slab with broken time‐reversal symmetry. Topological edge states in 2D periodic systems based on the quantum pseudo‐spin Hall effect have then been widely implemented.^[^ ^11^, ^16^, ^17^, ^18^, ^19^, ^20^, ^21^, ^22^ ^]^ In this last case, the propagation direction on the edges or interfaces is associated with a pseudo‐spin which can be related to the polarization of light^[^ ^16^, ^17^ ^]^ or a valley state.^[^ ^18^, ^19^, ^20^ ^]^ Quantum pseudo‐spin Hall effect is much easier to implement, the disadvantage is that the “protection” is only ensured if pseudo‐spin conversion is forbidden by a symmetry, which in practice is hardly realized against random disorder scattering. Beyond the existence and properties of edge states, the non‐trivial topology of bulk bands has also been used directly, through the excitation and study of valley‐polarized states in staggered honeycomb photonic crystals.^[^ ^23^ ^]^ Recently, the exciton‐polariton (simply called polaritons hereafter) in planar semiconductor microcavities^[^ ^24^, ^25^, ^26^ ^]^ have emerged as a new platform for topological photonics. Polaritons are hybrid bosons, originating from the strong coupling of excitons and cavity photons.^[^ ^27^, ^28^, ^29^ ^]^ The excitonic component of this symbiotic part‐light part‐matter particle allows strong interparticle interactions with resulting strong optical nonlinearities.^[^ ^29^ ^]^ Room temperature polariton operation such as polariton lasing can be achieved using large band gap semiconductors (GaN, ZnO)^[^ ^30^, ^31^ ^]^ or organics.^[^ ^32^, ^33^, ^34^, ^35^ ^]^

Polariton topological insulators have been predicted^[^ ^25^ ^]^ and observed^[^ ^36^ ^]^ at optical wavelength and low temperature. It is based on the use of a honeycomb lattice of coupled microcavity pillars and on the combination of photonic spin‐orbit coupling (SOC) based on the intrinsic chirality of transverse electric (TE) and transverse magnetic (TM) photonic modes^[^ ^37^, ^38^ ^]^ and excitonic Zeeman splitting^[^ ^39^ ^]^ which breaks the time‐reversal symmetry at optical wavelength. Another breakthrough first achieved in topological polaritonics was the concept of 1D topological lasers, which was proposed in refs. [40, 41] then observed in refs. [43, 44, 45] in the zero‐dimensional edge mode of a polaritonic Su‐Schrieffer‐Heeger chain. The field of topological lasers then strongly extended toward 2D systems with an initial proposal by Segev's group.^[^ ^42^ ^]^ Observation of 2D topological lasing occurring in topologically protected propagating edge modes was reported first in a system with broken time‐reversal symmetry,^[^ ^46^ ^]^ then through a quantum pseudospin‐like effect^[^ ^47^ ^]^ with preserved time‐reversal symmetry. After these seminal works, the field considerably extended toward the use of various platforms.^[^ ^44^, ^45^, ^48^, ^49^, ^50^, ^51^ ^]^ Another crucial possibility offered by microcavities is the direct measurement of wave functions and of the band geometry and topology.^[^ ^52^ ^]^ In bare 2D planar cavities, each confined mode forms a polarization doublet of massive quasi‐particles. TE‐TM SOC gives different masses to these two parabolas, which touch at k = 0, where they both carry Berry curvature monopoles of opposite charge (±1).^[^ ^37^, ^39^, ^53^ ^]^ Linear birefringence splits the two parabolas, which are now linearly crossing at 2 discrete points in the 2D k‐space, showing two tilted Dirac cones^[^ ^33^, ^53^, ^54^ ^]^ (optical valleys) each carrying topological charges ±1/2. In this case, excitonic Zeeman splitting can remove the degeneracy at the Dirac point, leading to the appearance of two valleys with the same circular polarization, same chirality, and same mass (gapped Dirac Hamiltonian).^[^ ^53^, ^55^ ^]^ Another way of removing the Dirac cones degeneracy while preserving the time‐reversal symmetry is by using an emergent optical cavity (also called Rashba–Dresshelhaus SOC with equal strength).^[^ ^33^, ^55^, ^56^ ^]^ In such a case, the two valleys show opposite chiralities (opposite Berry curvature and valley Chern number) and opposite circular polarization, as demonstrated experimentally in refs. [33, 55]. Such systems preserve the time‐reversal symmetry which leads to the trivial topology of the whole band, nevertheless, the nontrivial topology of the valleys has its practical applications. For example, the interface states are defined by the valley Chern number, which can be viewed as the “topology” of each single valley.

Despite the demonstration of various linear effects associated with structured band geometries, the interplay between the nonlinearities and geometrically rich band structures is still to be investigated.^[^ ^57^, ^58^, ^59^ ^]^ From the point of view of applications, circularly polarized lasers have attracted a great attention in nanophotonics,^[^ ^60^ ^]^ quantum optics,^[^ ^61^, ^62^ ^]^ and biophysics.^[^ ^63^, ^64^ ^]^ The reports on organic circularly polarized lasers mainly focus on organic chiral molecules^[^ ^65^ ^]^ or helical microstructures.^[^ ^66^ ^]^ However, there are some difficulties in the time‐consuming molecular synthesis and usually, single‐helicity laser emission was observed in a left‐handed or right‐handed helical material. Notably, topological wave guides naturally support helicity‐dependent light transport along separate one‐way channels.^[^ ^67^ ^]^ Obtaining circularly polarized lasing via the structured band geometry would be an interesting method that involves much easier process of material fabrication.

Here, we demonstrate helical polariton lasing from topological valleys in an organic anisotropic microcrystalline cavity. Valley states of opposite circular polarization and valley Chern number are formed as in ref. [33] by combined TE‐TM SOC, linear birefringence, and optical activity (OA). Polariton lasing occurs thanks to the use of a specific organic molecule, for which polaritonic gain was previously observed.^[^ ^32^ ^]^ Adjusting exciton‐photon detuning allows polariton lasing to occur specifically at the energy of the two valley states. Measuring the full 2D angular‐resolved emission pattern shows a strong anisotropy of lasing concentrated in the two valleys and shows a valley‐contrasted circular polarization degree, locked to the emission angle. Topological protection applies in our case to the circular polarization at the band extrema. Our findings provide key insights into helical topological polariton lasers and may lead to applications of topological organic laser devices in the strong coupling regime.

## 2 Result and Discussion

### 2.1 Selective Strong Coupling in an Organic Microcavity

**Figure 1:**
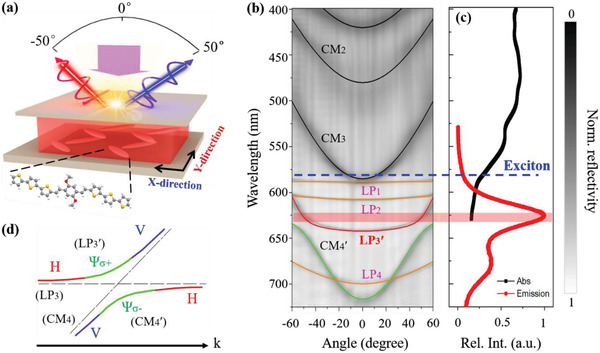
Schematics of organic microcavity setup and angle‐resolved reflectivity. a) Scheme of a TTPSB microbelt‐filled optical cavity sandwiched between two silver reflectors. b) Angle‐resolved reflectivity of the microcavity at room temperature. LP_n_ and CM_n_ denote the n‐th lower polariton (LP, orange lines) branch and V‐polarized cavity mode (CM, black lines), respectively. Because of the SOC interaction between LP_3_ and CM_4_ branches, two new hybrid branches of LP_3_′ (red line) and CM_4_′ (green line) are generated, as schematically shown in (d). c) The absorption (black line) and PL (red line) spectra of the TTPSB microbelt. d) Scheme of the mode splitting in the region of the anticrossing point of the V‐polarization mode (blue lines) and H‐polarization mode (red lines) with opposite parity. Two opposite circularly polarized modes (Ψ_*σ*+_ and Ψ_*σ*−_) are obtained due to the anticrossing.

Our organic microcavity consists of an organic active layer sandwiched between two metallic films as sketched in **Figure** **1**a. The active layer consists of single‐crystalline microbelts of an organic molecule, 1,4‐dimethoxy‐2,5‐di(2,2“,5”,2“”‐terthiophenestyryl)benzene (TTPSB). The details on the synthesis of TTPSB molecules, the preparation of microbelts, the fabrication of microcavities and their characterization are provided in the Supporting Information (see Experimental Section). In our case, the chosen microbelts have smooth surface and uniform morphology with typical width of around 50 µm, thickness of 720–730 nm and length of around several hundreds of micrometers (Figure S1, Supporting Information). These samples exhibit strong anisotropy along the axes of the microbelt crystal (i.e., X‐direction and Y‐direction) as shown in Figure 1a. We first analyze the unpolarized total reflectivity of the sample in X‐direction as shown in Figure 1b, where the reflectivity is plotted as a function of wavelength (or energy) and angle (or momentum k_x_), measured by using a home‐made angle‐resolved microscope at room temperature (see details in Supporting Information).

Two sets of modes with distinctive curvatures, marked respectively by black and orange lines, are observed (Figure 1b). We further performed polarization‐dependent angle‐resolved reflectivity of the same sample by adding a linear polarizer in the detection optical path. It turns out that these two sets of modes are orthogonally linearly polarized. For example, the modes with larger curvatures are horizontal (H)‐polarized (perpendicular to X‐direction), while the modes with smaller curvatures are vertical (V)‐polarized (parallel to X‐direction, see Figure 1a and Figure S2, Supporting Information). This is due to the strongly polarized nature of excitons in TTPSB microbelts, which is supported by the anisotropy of the excitonic absorption in these microbelts (Figure S3, Supporting Information). The excitons at the first excited singlet state (S_1_ at 587 nm) of TTPSB, as shown in the absorption spectrum (blue line in Figure 1c), can strongly couple only with the cavity photon modes that are H‐polarized, which are in very good agreement with the lower polariton (LP) dispersions calculated by using the coupled harmonic oscillator model (see Experimental Section in Supporting Information).^[^ ^68^ ^]^ In sharp contrast, the excitonic resonance at 587 nm does not affect at all the V‐polarized modes, which can be perfectly fitted by the planar cavity modes (Figure 1b,c). This is because the much stronger excitonic absorption for H‐polarized light (than that of V‐polarized one) benefits the photon‐exciton interaction, which causes the H‐polarized cavity photon modes can strongly couple with their vibronic replicas of TTPSB microbelt.

### 2.2 Anticrossing and Photoluminescence (PL) Spectra

More profoundly, the LP_3_′ (red line) and CM_4_′ (green line) modes as shown in Figure 1b anticross, form tilted Dirac cones in the vicinity of the angle *θ* = ± 50°, because of the recently discovered emergent OA,^[^ ^56^ ^]^ arising when two orthogonally polarized modes with opposite parity are tuned near resonance. It provides an effective Zeeman field, changing sign with wave vector. It lifts the original crossing modes (Figure 1b,d). Theoretically, the OA‐induced anticrossing can be efficiently described by an effective 2 × 2 Hamiltonian.^[^ ^33^ ^]^ However, the strong mediation of the exciton mode that results in the normal mode splitting (e.g., polaritons) can optimize the system's Hamiltonian, that is, a more accurate 4 × 4 Hamiltonian with the strong coupling of excitons and photons can be used to describe such a system. When the exciton mode strongly couples to only one of the orthogonally linearly polarized photon modes (H‐mode), the effective 4 × 4 Hamiltonian in the linear polarization basis in this scenario has the form

(1) $$\;H=\left(\begin{array}{cccc} E_{0,H} & 0 & \Omega /2 & 0\\ 0 & E_{0,V} & 0 & 0\\ \Omega /2 & 0 & E_{P,H}+\frac{\hbar^{2}k^{2}}{2m}+\beta_{0}\left(k_{x}^{2}-k_{y}^{2}\right) & -2i\beta_{0}k_{x}k_{y}+\xi k_{x}\\ 0 & 0 & 2i\beta_{0}k_{x}k_{y}+\xi k_{x} & E_{P,V}+\frac{\hbar^{2}k^{2}}{2m}-\beta_{0}\left(k_{x}^{2}-k_{y}^{2}\right) \end{array}\right)$$

**Figure 2:**
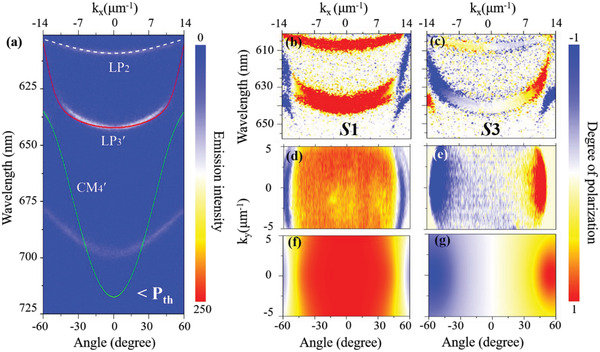
Angle‐resolved PL spectrum and Stokes parameters. a) Angle‐resolved PL spectrum measured upon excitation of a 400‐nm femtosecond laser at a low fluence. The LP_3_′ mode is marked by the red line and the CM_4_′ mode is marked by the green line. Measured Stokes parameters b) *S*_1_ and c) *S*_3_ of LP_2_ and LP_3_′ modes. 2D maps of the Stokes parameters d) *S*_1_ and e) *S*_3_ of the LP_3_′ mode extracted from Figure 2a. f,g) Theoretically calculated Stokes parameters corresponding to (d) and (e), respectively. The two solid lines in (a) and the numerical results in (f, g) are obtained by solving the Hamiltonian (1) with the parameters: *E*_0,*V*_ = *E*_0,*H*_ = 2.112 eV, *E*_*P*,*V*_ = 2.412 eV, *E*_*P*,*H*_ = 1.73 eV, Ω = 590 meV, *m* = 0.78 × 10^−4^ *m*_e_ (*m*_e_ is the free electron mass), *β*_0_ = 0.5 meV µm^2^, and *ξ* = 5.2 meV µm.

Here, *E*_0,*H*_ (*E*_0,*V*_) is the exciton energy in H‐ (V‐) polarization, *E*_*P*,*H*_ (*E*_*P*,*V*_) is the ground energy of the H‐ (V‐) polarized photon mode, Ω denotes the coupling of the cavity photon mode and the exciton mode, which occurs only in H‐polarization, *m* is the effective mass of cavity photons, *k* is the wavevector, *β*_0_ represents the TE‐TM splitting, and *ξ* describes the emergent OA. The two lower modes calculated from the Hamiltonian (1) agree very well with the experimental results, as shown in **Figures** 1b and **2**a. The two anticrossing regions correspond to tilted Dirac cones, a particular case of topologically nontrivial valleys exhibiting opposite Berry curvature, which is similar to our previous investigation.^[^ ^33^ ^]^ They are characterized by opposite valley Chern numbers equal to ±1/2.

As analyzed above, the linear polarizations of the two anticrossing modes are exchanged at higher wave vectors. Take the lower branch as an example: the linearly H‐polarized mode at a larger angle transforms into the linearly V‐polarized mode at a smaller angle across the right‐handed circularly polarized mode (Ψ_*σ*−_) (c.f. Figure 1d). Therefore, the linearly polarized photoluminescence (PL) can be emitted from the center of the reciprocal space, whereas oppositely circularly polarized emission should be detected in the anticrossing regions. In order to verify the validity of the anticipation, the angle‐resolved measurement of the PL spectra of the same sample has been carried out and shown in Figure 2a. Clearly, the PL emission distributes uniformly over the entire detected angle following the simulated polariton dispersions as indicated by the lines, evidencing that the angle‐resolved PL signals originate from the polariton emission. Here, the positions of LP_2_ and LP_3_′ emission spots match well with the spontaneous 0–0 and 0–1 PL transitions of the TTPSB microbelt, respectively (red line in Figure 1c). We further experimentally measured the Stokes vector of the signals (see details in Supporting Information). The *S*_1_ components of the Stokes vector of the LP_2_ and LP_3_′ branches show radically different polarization distributions (Figure 2b). The LP_2_ branch exhibits strong linearly polarized PL emission in the entire branch; however, the linear polarization of the LP_3_′ branch changes signs across the anticrossing regions. In comparison, the *S*_3_ component of the Stokes vector shows that there are very weak circularly polarized signals in the LP_2_ branch, whereas the circularly polarized signals in the LP_3_′ branch reach the maximal values at the anticrossing points and change sign at k_x_ = 0 because of the time‐reversal symmetry (Figure 2c). We also measured the 2D Stokes vector of the LP_3_′ branch in the reciprocal space (Figure 2d,e). It is clearly seen that in the *S*_1_ component the value is zero and changes signs around the anticrossing regions, while in the same regions in the *S*_3_ component the value reaches the maximum, but with opposite signs. We note that this kind of property occurs only along the k_x_ direction determined by the orientation of the optical axis of the material. These results are in very good agreement with the theoretical results in Figure 2f,g.

### 2.3 Polariton Lasing through Vibration‐Assisted Relaxation

It is well‐known that the photophysical properties of organic microcrystals are strongly influenced by their aggregation effects. Benefiting from their H‐aggregates (see the corresponding structural and spectroscopic analysis in Figures S4 and S5, Supporting Information), TTPSB microbelts exhibit good lasing gain behavior in general, because their large Stokes shift minimizes the self‐absorption, and the dipole‐allowed 0–1 emission transition facilitates the lasing emission process (Figure S6, Supporting Information). Importantly, the polaritons in the TTPSB crystal‐filled microcavity possess a large exciton fraction according to the fitting parameters of the LP_n_ (n = 1, 2, 3, and 4) branches at *θ* = 0° which corresponds to zero in‐plane wavevector (Table S3 and Figure S10, Supporting Information) due to the positive detunings between the cavity modes and the exciton mode, which facilitates the bosonic condensation.^[^ ^32^ ^]^

**Figure 3:**
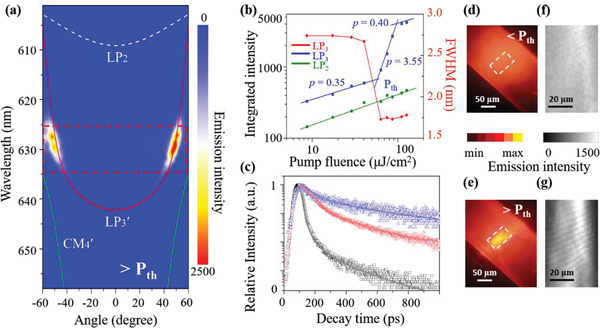
Polariton lasing emission. a) Angle‐resolved PL spectrum above the threshold at 125 µJ cm^−2^ (2.1 P_th_). b) Emission intensities of the LP_2_ (green) and LP_3_′ (blue) branches and the FWHM (red) of the LP_3_′ branch as a function of the pump fluence. c) PL decay profiles over time at different pump fluence: 11.9 (0.2 P_th_, blue), 35.7 (0.6 P_th_, red), and 71.5 µJ cm^−2^ (1.2 P_th_, black). Real‐space PL images recorded at d) 0.6 P_th_ and e) 1.2 P_th_. f,g) The corresponding spatial coherence images recorded by a Michelson interferometer.

In order to study the behavior of polariton lasing, the fluence of the pumping 400‐nm femtosecond laser is increased to above the threshold (P_th_) and the corresponding angle‐resolved PL spectrum is shown in **Figure** **3**a and Figure S7, Supporting Information. With the increase of the pump fluence to 89.4 µJ cm^−2^ (1.5 P_th_), the polariton intensity of LP_3_′ exhibits a sharp increase by about one order of magnitude near the two anticrossing points compared with that below the threshold, whereas the LP_2_ emission increases only a little (see Figure S8, Supporting Information). The clear power‐dependent blueshift of lasing spectra is visible beyond the P_th_ (Figure S9, Supporting Information). The blue line in Figure 3b shows the integrated PL intensity of LP_3_′ emission as a function of the pump fluence, showing a typical threshold curve. The intensity dependence is separately fitted to power laws *x^p^* with *p* = 0.35 ± 0.01, 3.55 ± 0.04, and 0.40 ± 0.02, respectively. The threshold of the polariton lasing is P_th_ = 59.6 µJ cm^−2^ at the first intersection between the sublinear and superlinear regions. Meanwhile, the full width at half‐maximum (FWHM) of the LP_3_′ emission (red line) dramatically narrows from 2.75 nm below the threshold to 1.75 nm above the threshold. In sharp contrast, the integrated PL intensity of the LP_2_ emission presents a linear and slow increase (green line), indicating that the polaritons in the LP_2_ branch remain in the spontaneous emission regime with unchanged FWHM. Figure 3c shows the time‐resolved PL from the LP_3_′ branch measured by a streak camera. At a very low pump density of 11.9 µJ cm^−2^ (0.2 P_th_), the polariton PL follows a single‐exponential decay with a lifetime of *τ* = 0.36 ± 0.01 ns. Upon increasing the pump density, a bi‐exponential decay is observed, with the short component ascribed to bimolecular quenching.^[^ ^69^ ^]^ Above the threshold, the PL decay time collapses to less than 30 ps, indicating the bosonic stimulation of the relaxation process,^[^ ^70^ ^]^ and is limited by the resolution of our apparatus. All the above features confirm the polariton lasing at the LP_3_′ branch. The exact coincidence of the LP_3_′ branch with the 0–1 transition of TTPSB microbelts further suggests that the vibration‐assisted relaxation mechanism selectively populates the LP_3_′ branch from the exciton reservoir through emitting a vibron.^[^ ^32^, ^71^ ^]^ In our case, the anti‐crossing point generated by the coupling of the H‐ and V‐polarized modes also form a curvature‐inflection point, which slows down polariton relaxation. Therefore, due to the anti‐crossing point and the lower quality factor of our cavity (Q ≈ 300), the polariton lasing does not occur at the ground state of the LP_3_′ branch, but rather in the anticrossing region playing the role of the bottleneck.

The long‐range spatial coherence is also an important defining feature for polariton lasing. To demonstrate it, we sent the real‐space PL image into a Michelson interferometer, in which one arm is a retroreflector to invert the PL image in a centro‐symmetrical configuration. When the PL emission image and its inverted one are superimposed, no clear interference fringes can be observed at the pump fluence of 35.7 µJ cm^−2^ (0.6 P_th_) (Figure 3d), representing the spontaneous regime. However, when the pump fluence exceeds the threshold, clear interference fringes can be well resolved throughout the whole detection region (Figure 3e), confirming that the long‐range spatial coherence is established in this case.

### 2.4 Helical Polariton Lasing from Topological Valleys

**Figure 4:**
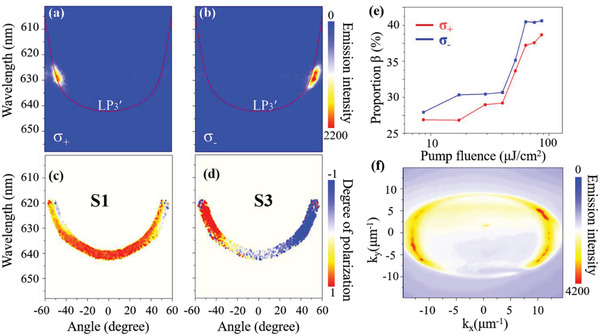
Circular polarization distribution (*σ*_+_ and *σ*_−_). Angle‐resolved PL spectra with a) left‐handed and b) right‐handed circular polarization above the threshold. Measured Stokes parameters c) *S*_1_ and d) *S*_3_ of the polariton laser from the LP_3_′ branch, corresponding to (a) and (b), respectively. e) Circularly polarized proportion *β* of the output laser as a function of the pump fluence. f) Emission intensity in 2D reciprocal space above the lasing threshold, demonstrating the occupation of the polaritons in the two topological valleys.

To understand the polarization properties of the polariton laser, we further performed the circularly polarized (*σ*+ and *σ*−) angle‐resolved PL spectroscopy. Expectedly, the polariton lasing emission exhibits strong left‐handed circular polarization in the vicinity of one anticrossing point of about −50° (**Figure** **4**a), whereas a right‐handed circularly polarized polariton laser can be obtained in the vicinity of the other anticrossing point of about +50° (Figure 4b). We further measured the Stokes vector, and it shows that in the S3 component the maximal polarization degree is observed in the vicinity of two anticrossing points (Figure 4d). The parameter which is topologically protected, namely robust against weak perturbations of the effective Hamiltonian, is the circular polarization of the band extrema in the two valleys which are 100% circularly polarized. Direct disorder scattering from one valley to another cannot occur as well, because it has to preserve polarization. On the other hand, scattering toward other states of the dispersion is possibly providing a channel for losses.

It is worth noting that the luminescence dissymmetry factor (*g*_lum_) of our polariton laser is as high as 1.90, according to the equation, *g*_lum_ = 2 × (I_*σ*+_ − I_*σ*−_)/(I_*σ*+_ + I_*σ*−_), where I_*σ*+_ and I_*σ*−_ correspond to the laser output of left‐ and right‐handed polarization, respectively.^[^ ^72^ ^]^ This highly dissymmetric *g*_lum_ stemming from topological valleys is far exceeding the reported circularly polarized light from organic systems, which may offer a new strategy for amplifying the chirooptical response of organic small emissive molecules. Figure 4e shows the circularly polarized proportion *β* (defined as the ratio between the intensity emitted from the valleys I_*σ*+_ or I_*σ*−_ and the total intensity) of the laser output as a function of the pump fluence.

(2) $$\beta =\frac{\mathrm{Emission}\;{\mathrm{Intensity}}_{\sigma +/\sigma -}}{\frac{1}{2}\times \mathrm{Overall}\;\mathrm{Emission}\;\mathrm{Intensity}}$$

Interestingly, *β* values exhibit a significant increase from 27% to 41% for *σ*+ and from 26% to 39% for *σ*−. Clearly, the nonlinear condensation process has brought out the significantly enhanced helical characteristics of the output light. This prominent chirality amplification benefits from the rapid population of polaritons into the anticrossing regions in the polariton lasing process. Figure 4f shows the emission in 2D reciprocal space above the lasing threshold. It is clear that the lasing emission mainly focuses in the vicinity of the anticrossing points, which demonstrates the occupation of the polaritons in the two topological valleys.

## 3 Conclusions

In conclusion, we demonstrate helical topological polariton lasers in an organic anisotropic microcrystalline cavity by strongly coupling Frenkel excitons with the local nontrivial band geometry. Experimental and theoretical results indicate that the oppositely circularly polarized polariton lasers emit along different angular directions. The strong interaction of the excitonic component comes into play in bosonic condensation and the photonic component offers topological valleys. The left‐handed and right‐handed circularly polarized lasing is topologically protected in the sense that the band extrema in the valleys are guaranteed to be 100% circularly‐polarized. The significantly enhanced helical characteristics of output light are achieved by the nonlinear condensation process. Asymmetric emission between the two opposite circular polarizations is possible to be achieved by further breaking the time‐reversal symmetry, most probably via the Zeeman effect of excitons by applying an external magnetic field. Helical topological polariton lasers might provide a platform for the exploration of topological phenomena involving light‐matter interaction and the development of polariton‐based spintronic devices.

## Conflict of Interest

The authors declare no conflict of interest.

## Authors Contribution

T.L., J.‐H.R., and Q.L. designed the experiments and performed experimental measurements. X.M., S.S., G.M., and D.S. performed the theoretical calculation and analysis. T.L., X.M., F.L., Q.L., S.S., G.M., D.S., and H.‐B.F. wrote the manuscript with contributions from all authors. Q.L. and H.‐B.F. supervised the project. All authors analyzed the data and discussed the results.

## Supporting information

Supporting Information

## Data Availability

Research data are not shared.
