# Supplementary material for: Helical Polariton Lasing from Topological Valleys in an Organic Crystalline Microcavity
Source: Adv Sci (Weinh). 2022 Aug 21;9(29):2203588. doi: 10.1002/advs.202203588 (PMC9561778; doi:10.1002/advs.202203588)
Supplement: Supplementary file 1 — Supporting Information [file ADVS-9-2203588-s001.pdf]

ADVANCED SCIENCE
Open Access

Supporting Information

for *Adv. Sci.*, DOI 10.1002/advs.202203588

Helical Polariton Lasing from Topological Valleys in an Organic Crystalline Microcavity

Teng Long, Xuekai Ma, Jiahuan Ren, Feng Li, Qing Liao, Stefan Schumacher, Guillaume Malpuech, Dmitry Solnyshkov and Hongbing Fu*

Supporting Information

Helical polariton lasing from topological valleys in an organic crystalline microcavity

Teng Long, Xuekai Ma, Jiahuan Ren, Feng Li, Qing Liao, Stefan Schumacher, Guillaume Malpuech, Dmitry Solnyshkov, Hongbing Fu*

T. Long, Prof. Q. Liao, Prof. H. B. Fu
Beijing Key Laboratory for Optical Materials and Photonic Devices, Department of Chemistry, Capital Normal University, Beijing 100048, People's Republic of China
E-mail: liaoqing@cnu.edu.cn

Dr. X. Ma, Prof. S. Schumacher
Department of Physics and Center for Optoelectronics and Photonics Paderborn (CeOPP), Universität Paderborn, Warburger Strasse 100, 33098 Paderborn, Germany

J. H. Ren
Tianjin Key Laboratory of Molecular Optoelectronic Science, School of Chemical Engineering and Technology, Collaborative Innovation Center of Chemical Science and Engineering (Tianjin), Tianjin University, Tianjin 300072, P. R. China

Prof. F. Li
Key Laboratory for Physical Electronics and Devices of the Ministry of Education & Shaanxi Key Lab of Information Photonic Technique, School of Electronic Science and Engineering, Faculty of Electronic and Information Engineering, Xi'an Jiaotong University, Xi'an 710049, China

Prof. S. Schumacher
Wyant College of Optical Sciences, University of Arizona, Tucson, Arizona 85721, United States

Prof. G. Malpuech, Prof. D. Solnyshkov
Institut Pascal, PHOTON-N2, Université Clermont Auvergne, CNRS, Clermont INP, F-63000 Clermont-Ferrand, France

Prof. D. Solnyshkov
Institut Universitaire de France (IUF), 75231 Paris, France

MATERIALS AND METHODS

1. Synthesis of TTPSB

The compound used in our work, 1,4-dimethoxy-2,5-di(2,2',5',2''-ter-thiophenestyryl) benzene (TTPSB) was synthesized according to Horner-Wadsworth-Emmons reaction (F. Gao *et al.*, *Angew. Chem. Int. Ed.* 2010, 49, 732-735. & Z. Xu *et al.*, Adv. Mater. 2012, 24, OP216-OP220.). All starting materials were purchased from Alfa Aesar and used as received without further purification. The tetrahydrofuran (THF, HPLC grade) and hexane were purchased from Beijing Chemical Agent Ltd., China. Ultra-pure water with a resistance of 18.2 MΩ·cm^{-1} were used in all experiments, produced by Milli-Q apparatus (Millipore).

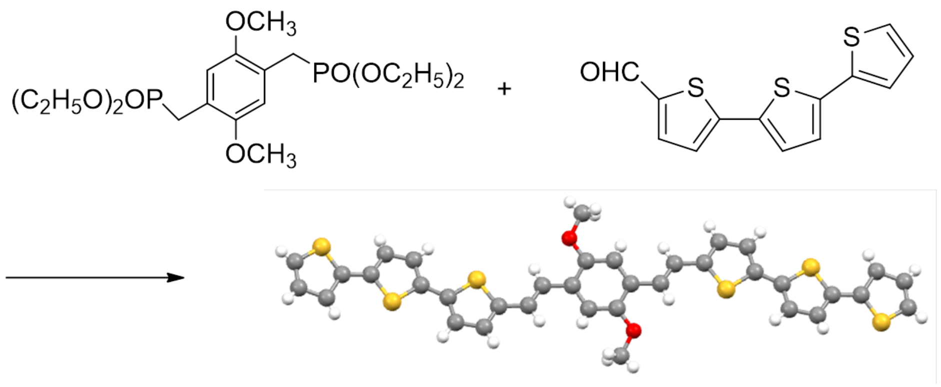

Scheme S1. The synthesis of the TTPSB molecule.

Without water and oxygen, a mixture of 2,5-bismethoxy-1,4-xylene-bis(diethyl phosphonate) (510 mg, 1.16 mmol) and catalyzer NaH (54.72mg, 2.28mmol) in tetrahydrofuran (THF) solution was cooled in an ice bath at 0 °C during a 30 min period. Then the 2, 2'-Bithiophene-5-carboxaldehyde (620.0 mg, 2.26 mmol)/THF solution inject reaction bulb. (Scheme S1) And the reaction mixture was stirred at room temperature for 6 hours and subsequently poured into a little water, spin dry.

Then, pouring the orange powders into the Buchner funnel and wash it with alcohol. Finally orange powder was obtained as the compound (425.2 mg, 0.62 mmol) in 53% yield. 1H NMR (400 MHz, C4D8O): δ 7.41-7.32 (m, 4 H), 7.28-7.22 (m, 4H), 7.2 (s, 2H), 7.18-7.13 (m, 6 H), 7.04-7.01 (m, 4H), 3.91 (s, 6 H); MS (MALDI-TOF): 682.0.

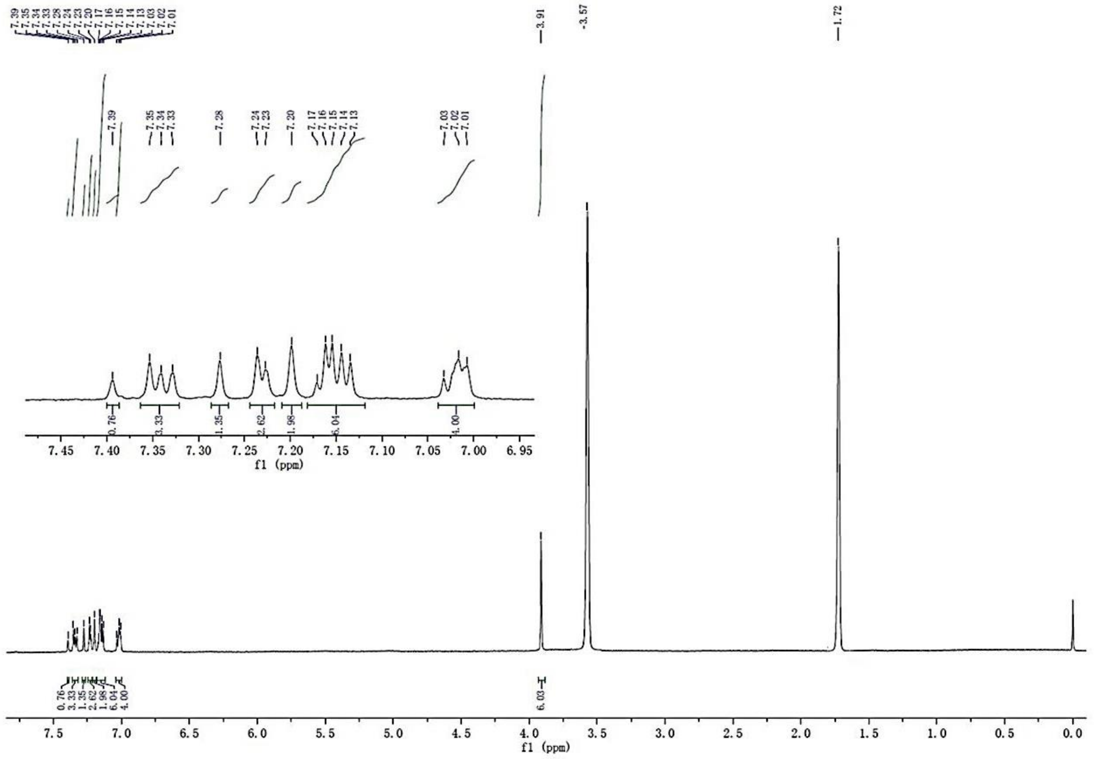

[1]H Nuclear magnetic resonance（NMR) spectrum of TTPSB.

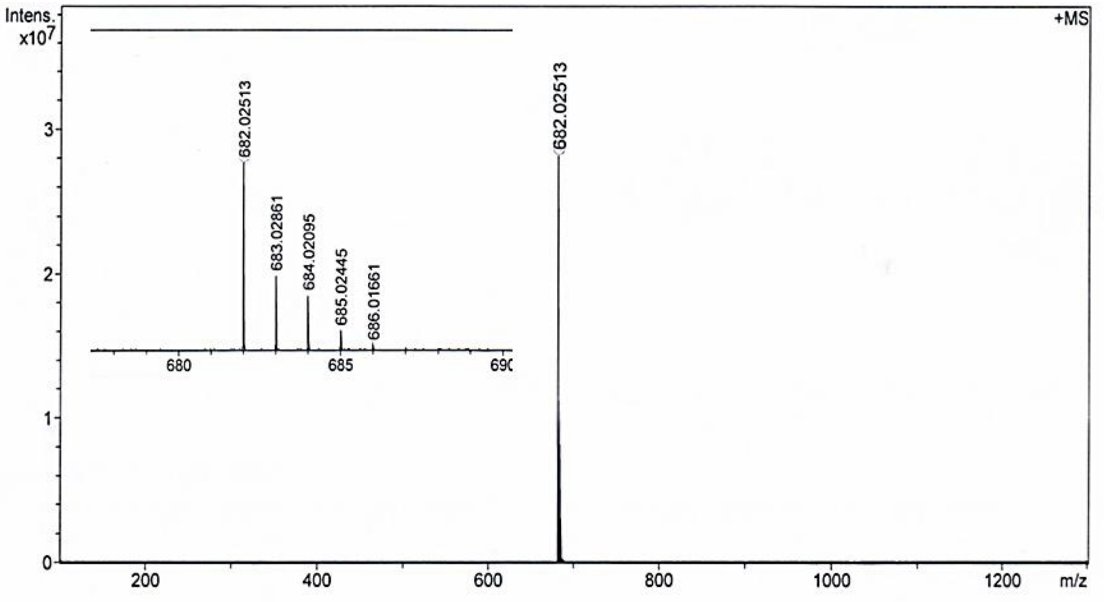

MALDI-MS spectrum of TTPSB.

2. The preparation of TTPSB microbelts

In our experiment, TTPSB microbelts were fabricated using a facile physical vapor deposition (PVD) method. A quartz boat carrying 3 mg TTPSB was then placed in the center of a quartz tube which was inserted into a horizontal tube furnace. A continuous flow of cooling water inside the cover caps was used to achieve a temperature gradient over the entire length of the tube. To prevent oxidation of TTPSB, Ar was used as inert gas during the PVD process (flowrate: 15 $sccm \cdot min^{-1}$). The pre-prepared hydrophobic substrates were placed on the downstream side of the argon flow for product collection and the furnace was heated to the sublimation temperature of TTPSB (at temperature region of ~ 320 °C), upon which it was physically deposited onto the pre-prepared hydrophobic substrates at temperature region of ~ 230 °C for 1 hours.

3. The preparation of TTPSB microcavity

Firstly, we use the metal vacuum deposition system (Amostrom Engineering 03493) to thermally evaporate silver film with the thickness of 85 ± 5 nm (reflectivity R ≥ 99%) on the glass substrate, the root mean square roughness (Rq) of the silver film in the 5 μm × 5 μm area is 2.45 nm, a 20 ± 2 nm SiO_2 layer was deposited using vacuum electron beam evaporate on the silver film with R_q of 2.31 nm, the deposited rates were both 0.2 Å/s and the base vacuum pressure is 3×10^{-6} Torr. This silver/SiO_2 film composite structure was placed as a substrate in a horizontal tube furnace for sample deposition. The TTPSB microbelts were uniformly dispersed on the silver/SiO_2 film substrate. Then 20 ± 2 nm SiO_2 and 35 ± 2 nm (R ≈ 50%) silver was fabricated to

form the microcavity. The 20-nm SiO_2 layers is used to prevent the fluorescence quenching of TTPSB microbelts caused by directly contact of the metallic silver with the crystal.

4. Structural and spectroscopic characterization

As-prepared TTPSB microbelts were characterized by field emission scanning electron microscopy (FE-SEM, HITACHI S-4800) by dropping on a silicon wafer. Samples examined by transmission electron microscopy (TEM, JEOL, JEM-2100) were obtained by one drop of the solution being dropped on a carbon-coated copper grid and evaporated. TEM measurement was performed at room temperature at an accelerating voltage of 100 kV. The X-ray diffraction (XRD, Japan Rigaku D/max-2500 rotation anode X-ray diffractometer, graphite monochromatized Cu K_α radiation (λ =1.5418 Å)) operated in the 2θ range from 3 to 30°, by using the samples on a cleaned glass slide.

The fluorescence micrograph, diffused reflection absorption and emission spectra were measured on Olympus IX71, HITACHI U-3900H, and HITACHI F-4600 spectrophotometers, respectively. Fluorescence quantum yield (Φ) of TTPSB monomer solution in THF measured through a relative method by using Rhodamine 6G as a standard and Φ of TTPSB microbelts measured through an absolute method by using an integration sphere. The photoluminescence spectra of isolated single TTPSB microbelt in microcavity was characterized by using a homemade optical microscope equipped with a 50 × 0.9 NA objective (Scheme S2). The second harmonic (λ = 400 nm, pulse width 150 femtosecond) of a 1 kHz Ti: sapphire

regenerative amplifier was focused to a 50-μm diameter spot to excite the selected single TTPSB on a two-dimensional (2D) movable table. Spatially resolved PL spectra were collected underneath by using a 3D-movable objective and detected using a liquid-nitrogen cooled charge-coupled device (CCD).

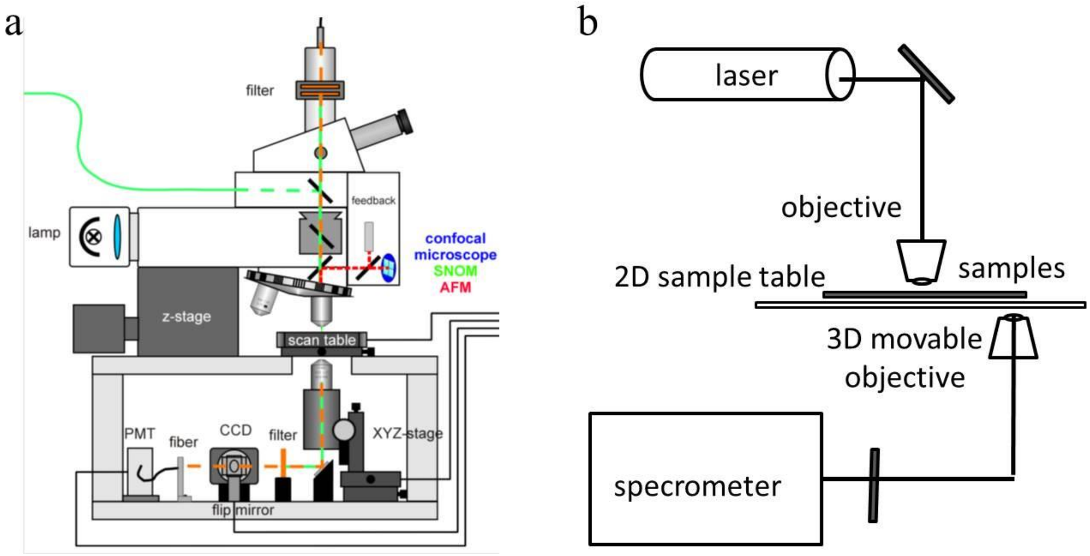

Scheme S2. Schematic demonstration of the experimental setup for the optical characterization: (a) the near-field scanning optical microscopy, and (b) the transmittance optical path for the waveguide measurements.

5. The angle-resolved spectroscopy characterization

The angle-resolved spectroscopy was performed at room temperature by the Fourier imaging using a 100× objective lens of a NA 0.95, corresponding to a range of collection angle of ±60° (Scheme S3). An incident white light from a Halogen lamp with the wavelength range of 400-700 nm was focused on the area of the microcavity containing a TTPSB microbelt. The k-space or angular distribution of the reflected light was located at the back focal plane of the objective lens. Lenses L1-L4 formed a

confocal imaging system together with the objective lens, by which the k-space light distribution was first imaged at the right focal plane of L2 through the lens group of L1 and L2, and then further imaged, through the lens group of L3 and L4, at the right focal plane of L4 on the entrance slit of a spectrometer equipped with a liquid-nitrogen-cooled CCD. The use of four lenses here provided flexibility for adjusting the magnification of the final image and efficient light collection. Tomography by scanning the image (laterally shifting L4) across the slit enabled obtaining spectrally resolved two-dimensional (2D) k-space images.

In order to investigate the polarization properties, we placed a linear polarizer, a half-wave plate and a quarter-wave plate in front of spectrometer to obtain the polarization state of each pixel of the k-space images in the horizontal-vertical (0° and 90°), diagonal (±45°) and circular (σ+ and σ−) basis (S. Dufferwiel *et al.*, *Phys. Rev. Lett.* 2015, 115, 246401. & F. Manni *et al.*, *Nat. Commun.* 2013, 4, 2590.). Thereby, one can calculate the Stokes vector through:

$$S_1 = \frac{I_{0^\circ} - I_{90^\circ}}{I_{0^\circ} + I_{90^\circ}}$$

$$S_3 = \frac{I_{\sigma+} - I_{\sigma-}}{I_{\sigma+} + I_{\sigma-}}$$

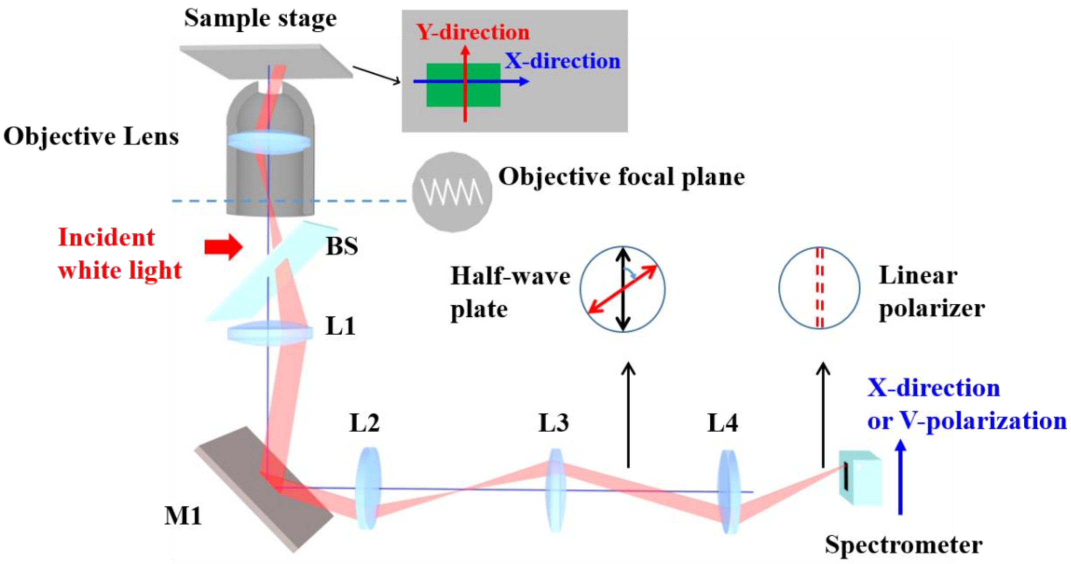

Scheme S3. Experimental setup allowing to obtain polarization-resolved complete state tomography. BS: beam splitter; L1-L4: lenses; M1: mirror. The red beam traces the optical path of the reflected light from the sample at a given angle.

The reflectivity measurement of our setup is shown below (Scheme S4). The reflectivity was also measured using a Halogen lamp with wavelength range of 400-700 nm. The light source was entered (green and white lines) and collected by using the same 100× microscope objective with a high numerical aperture (0.95 NA). The measurement angle can be achieved between ±60°. The pink and white lines in the figure below indicate the excited light paths.

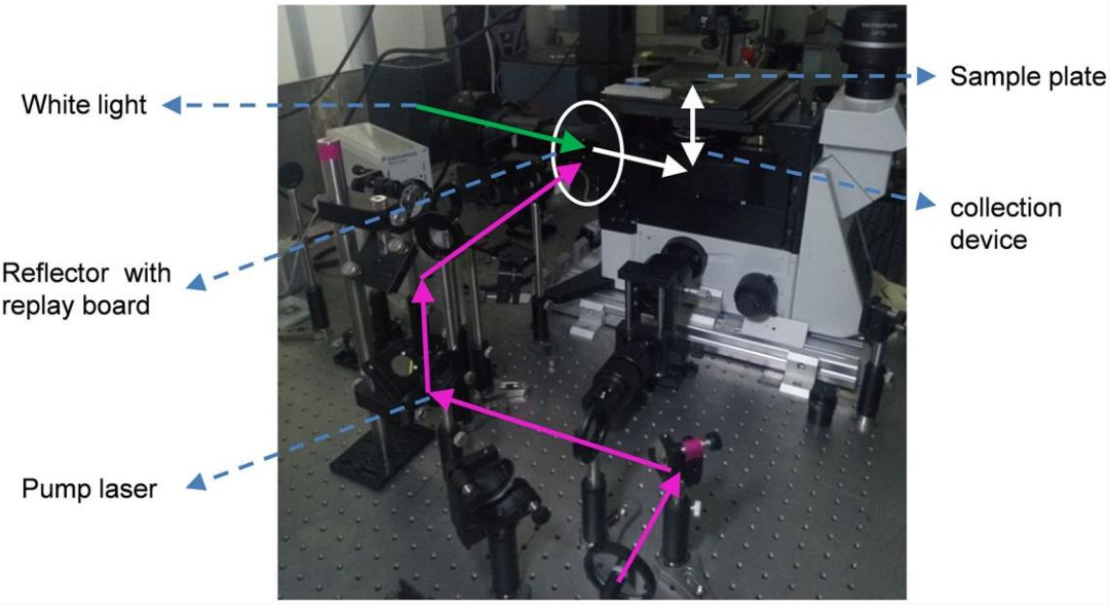

Scheme S4. Picture of our home-made experimental setup.

The reflectivity and photoluminescence spectroscopy were detected at room temperature in a home-made micro-area Fourier image which is presented to the spectrometer slit through four lenses. The schematic of the angle-resolved experimental setup is shown in Scheme S3. The reflected spectrum was collected by the spectrometer with a 300 lines/mm grating and a 400×1340 pixel liquid nitrogen cooled charge-coupled device (CCD). For off-resonant optical pumping (400 nm, pulse width 150 fs) from a 1 kHz Ti: sapphire regenerative amplifier and 40-μm spot diameter with a near Gaussian beam profile.

6. Polariton dispersion

The polariton dispersion in Fig. 1b was calculated by a coupled harmonic oscillator Hamiltonian (CHO) model (S. Kena-Cohen *et al.*, *Phys. Rev. Lett.* 2008, 101, 116401.). The 2×2 matrix in equation (1) below describes the CHO Hamiltonian:

$$\begin{pmatrix} E_{CMn}(\theta) & \Omega/2 \\ \Omega/2 & E_X \end{pmatrix} \begin{pmatrix} \alpha \\ \beta \end{pmatrix} = E \begin{pmatrix} \alpha \\ \beta \end{pmatrix} \tag{1}$$

Where θ represents the polar angle, $E_{CMn}(\theta)$ is the cavity photon energy of the nth cavity mode as a function of θ, E_X is the exciton 0–0 absorption energy of TTPSB microbelts at 2.11 eV (587 nm) and Ω (eV) denotes the coupling. The magnitudes $|\alpha|^2$ and $|\beta|^2$ correspond to the photonic and the excitonic fraction, respectively.

The cavity photon dispersion is given by:

$$E_{\mathrm{CMn}}(\theta) = \sqrt{\left(E_c^2 \times \left(1 - \frac{\sin^2\theta}{n_{eff}^2}\right)^{-1}\right)} - (n-1) \times l \qquad (2)$$

where E_c represents the cavity modes energy at θ = 0°, $E_{CM1}(\theta)$ represents the energy of the first cavity mode when n=1, $(n-1)\times l$ represents the energy difference from the first cavity mode. The effective refractive index (n_{eff} = 1.8 and 3) is extracted from the fitting results. The theoretical fitting dispersion of the uncoupled cavity modes (n_{eff} = 1.8, black solid line) and coupled cavity modes (n_{eff} = 3, black dash line) is shown in Figure 1b. Diagonalization of this Hamiltonian yields the eigenvalues, $E_{\pm}(\theta)$, which represents the upper and lower polariton (UP and LP) in-plane dispersions (H. Deng *et al.*, *Rev. Mod. Phys.* 2010, 82, 1489-1537.),

$$E_{\pm}(\theta) = \frac{E_X + E_{\mathrm{CMn}}(\theta)}{2} \pm \frac{1}{2}\sqrt{\left(E_X - E_{\mathrm{CMn}}(\theta)\right)^2 + \hbar^2\Omega^2} \qquad (3)$$

Crystal characterization

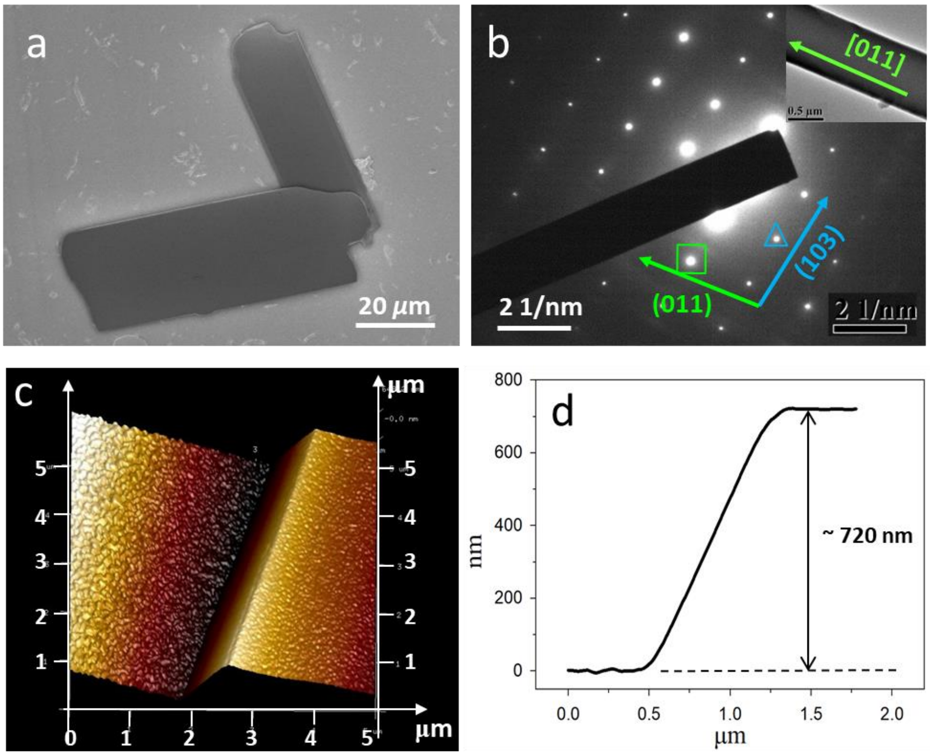

Figure S1. (a) Scanning electron microscopy (SEM) image of as-prepared TTPSB microbelts, which shows that the chosen microbelt has a typical length of about 50 μm. (b) Selected area electron diffraction (SAED) pattern recorded by directing the electron beam perpendicular to the top-surface of a single TTPSB microbelt. Inset: the corresponding transmission electron microscopy (TEM) image. These reveal that the TTPSB microbelt is single crystalline. (c) Atomic force microscopy (AFM) image of as-prepared TTPSB microbelts. (d) The corresponding topography line profile measured across the microbelt, which indicates that the chosen microbelt has a typical thickness of 720-730 nm.

Scanning and transmission electron microscopy (SEM and TEM) depict that typical TTPSB microbelts with smooth surface and sharp edge (inset in Figure S1a) have been successfully fabricated on a large scale (Figure S1a). Their length (*l*) is found to

range between 40 and 60 μm, while the width (*w*) and the height (*h*) is determined to be about 40 μm and 720 nm, respectively, according to atomic force microscopy (AFM) measurements (Figure S1c and d). The observed sharp spots in selected area electron diffraction (SAED) pattern (Figure S1b) clarifies that these microbelts are single crystalline in nature. According to that monoclinic TTPSB crystals belong to the space group of P21/n, with cell parameters of a = 10.4006 Å, b = 5.5454 Å, c = 27.0574 Å, α = γ = 90°, and β = 93.409° (CCDC No. 1554859, Table S1), the blue triangle spot in Figure S1b is ascribed to (103) Bragg reflections with a *d*-spacing value of 13.558 Å, and the red square spot corresponds to (011) crystal plane with *d*-spacing value of 11.328 Å, in good agreement with the cell parameters of this monoclinic crystal structure.

Table S1. Crystal data and structure refinement for TTPSB.

Space Group	P 2_1/n
a	10.4006Å
b	5.54540Å
c	27.0574Å
α	90.000º
β	93.409º
γ	90.000º

Orthogonal linear polarization angle-resolved reflectance spectrum

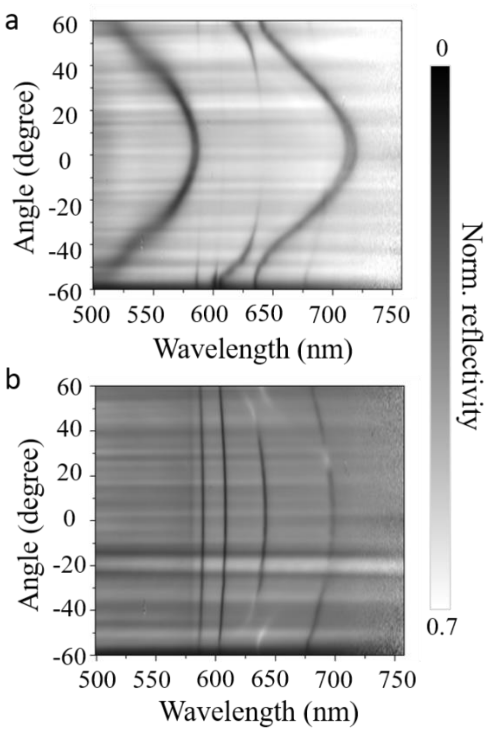

Figure S2. Measured k-space angle-resolved reflectivity spectra of a selected microcavity at room temperature in vertical (V) polarization (a) and horizontal (H) polarization (b) along X-direction of the single-crystal cavity.

Polarization-dependent absorption of the pure crystal

A broad and intense absorption peak is observed (blue line) when the polarization of the white light from a halogen lamp is adjusted to be perpendicular to the X-direction of the microbelt, whereas the absorption is much weaker (red line) when the polarization of the white light is parallel to the X-direction of the microbelt. The descriptions of polarization directions also see Supplementary Scheme S3. These distinct polarization-dependent absorption features are consistent with the fact that the anisotropy is a result of the highly ordered uniaxial alignment of TTPSB molecules in single crystalline microbelts.

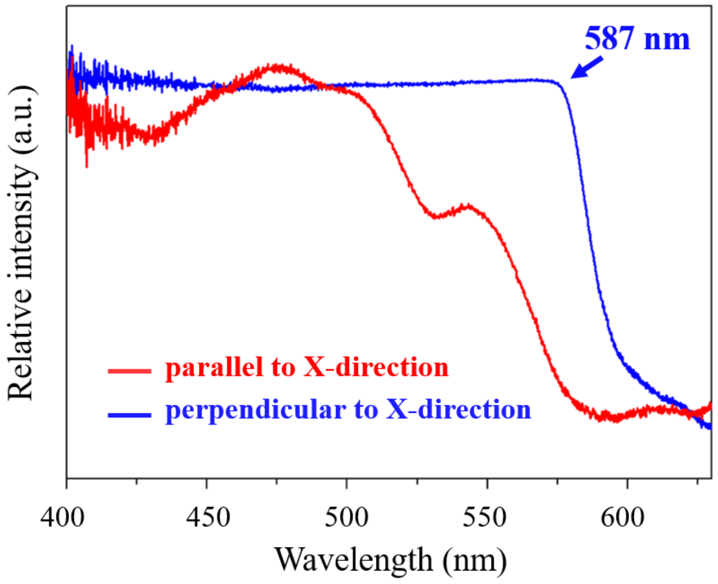

Figure S3. Polarization-dependent absorption spectra of a single TTPSB microbelt.

Crystal structure and simulation

It can be seen from Figure S4a that X-ray diffraction (XRD) spectra (red line) of ensemble microbelts deposited on a silicon substrate is dominated by the (002) and (10-3) series of peaks compared to XRD spectrum of TTPSB powder (black line), suggesting that these crystal facets are the main exposed surfaces of microbelts. Taking into account TEM image (inset of Figure S1b), it can be concluded that TTPSB microbelts grow preferentially along the crystal [011] direction. We also simulated the growth shape of TTPSB crystal based on the attachment energies using Material Studio package (D. Winn *et. al*, *AIChE J.* 2000, 46, 1348-1367.). We found that the predicted thermodynamic stable morphology is also belt-like structure, and the preferential growth along the crystal [011] direction is also the main stacking direction predicted (Figure S4b), which agrees with the results of the observed 1D microbelts (Figure S1a). According to Kasha's exciton model (M. Kasha, *Discuss. Faraday Soc.* 1950, 9, 14-19.), intermolecular herringbone packing generally advocates H-aggregation.

Combining the analysis of SAED, TEM and XRD results, TTPSB molecules within 1D microbelts adopt a herringbone packing arrangement and stack co-facially along the crystal [011] plane with the shortest separation about 3.47 Å, which indicates a typical π-π stacking (Figure S4c). Further analyze the molecules arrangement (Figure S4d-g), the pitch angle (which defines the angle between the molecular transition dipole and the π-stack direction) of 82.2° (corresponding to the longitudinal displacements between neighboring molecules of 0.47 Å) ensures co-facially

π-stacking, which might be beneficial to efficient charge transport channel for 1D-MWs. While the roll angle of 39.3° (corresponding to the transverse displacements 4.23 Å) greatly reduces the quenching effect caused by the π-π interaction and brings strong PL emission. According to the molecular exciton model (F. Würthner *et. al*, *Angew. Chem. Int. Ed.* 2011, 50, 3376-3410.), the pitch angle is <54.7° in a J-type aggregate and >54.7° in an H-type aggregate. Therefore, it is expected that H-type coupling occurs in TTPSB microbelts.

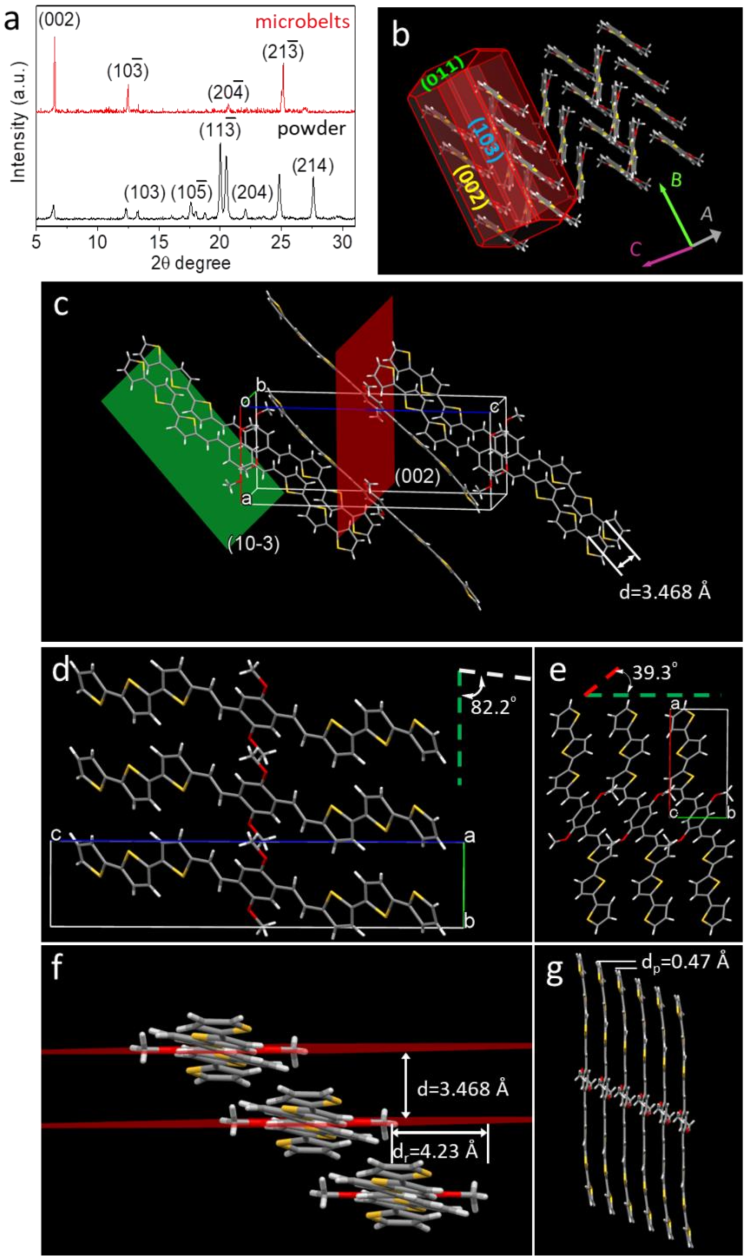

Figure S4. (a) XRD profiles of microbelts (red line) and powder (black line). (b) Simulated growth morphology of TTPSB molecules using Material Studio package. (c) Molecular packing arrangement of TTPSB in the microbelts, viewed almost along the crystal *b*-axis. (d) Viewed normal to *bc* plane with illustration of pitch angle. (e) Viewed normal to *ab* plane with illustration of roll angle. (f) Viewed the shortest separation of d = 3.468 Å and the transverse displacement of d_r = 4.23 Å. (g) Viewed the longitudinal displacement of d_p = 0.47 Å.

Photophysical and photochemical characterization of molecules and crystals

Figure S5 depicts the absorption and photoluminescence (PL) spectra of TTPSB monomers in diluted tetrahydrofuran (THF) solution and ensemble microbelts placed on a quartz substrate. The related photophysical parameters were summarized in Table S2. The absorption spectrum of the diluted solution exhibits a broad featureless peak at 473 nm attributed to the lowest $S_0 \rightarrow S_1$ transition of the monomers. PL emission shows clearly vibrationally structure at 538, 567, and 611 nm with contributions arising mainly from vinyl stretching mode (top panel of Figure S5a). In intense contrast, the maximum absorption spectrum of microbelts shows a slight blue-shift to 470 nm, with additional bands around 543 nm (2A) and 566 nm (1A) due to aggregate states in microbelts (bottom panel of Figure S5a). Their PL spectrum is dominated by 0-1 transition and maximum emission posits at 621 nm, due to the self-absorption caused by the overlap between their absorption and their emission. This is a specific fingerprint of H-type aggregates with a "face-to-face" molecular-packing arrangement.

TTPSB monomers has poorly emission with the PL quantum yield (Φ) of 0.04 ± 0.01 through a relative method by using Rhodamine 6G as a standard, while microbelts exhibit a moderate value Φ of 0.15 ± 0.01 through an absolute method by using an integration sphere (Table S2). This is a typical "aggregation-induced emission (AIE)". To further investigate the nature of the excited states, we performed time-resolved fluorescence measurements by single photon counter (Figure S5b and S5c). The monomer solution emission at 540 nm decays monoexponentially, yielding

a lifetime of τ_m = 182 ± 1.4 ps. The PL decay of microbelts at 625 nm was also fitted monoexponentially with a time constant of $\tau_{microbelts}$ = 425 ± 2.3 ps, which is much longer than that of TTPSB monomers. Based on the equation of $k_r = \Phi/\tau$ (M. Gsänger *et. al*, *Angew. Chem. Int. Ed.* 2010, 49, 740-743.), the radiative decay rates (k_r) are calculated to be $k_{r,m}$ = 0.220 ns^{-1} and $k_{r,microbelts}$ = 0.353 ns^{-1} for monomers and microbelts, respectively (Table S2). The restriction of rotation motion of terthiophenestyryl substituents in solid state were considered to enhance the nonradiative decay and result in AIE effect.

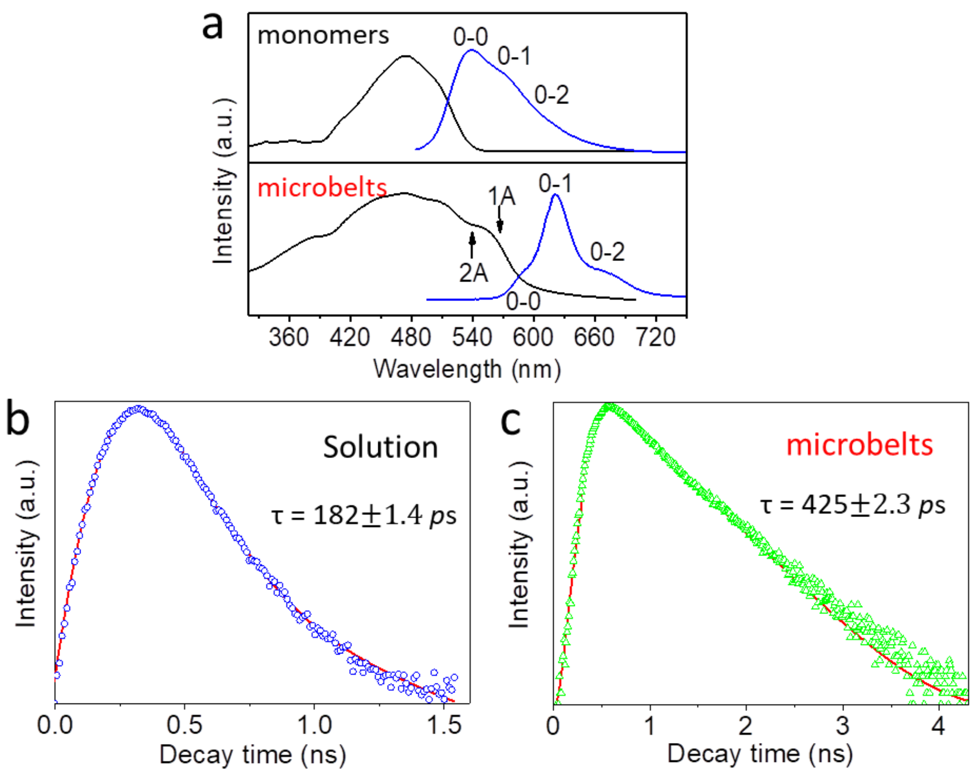

Figure S5. (a) Normalized absorption (black line) and PL (blue line) spectra of ensemble microbelts placed on a glass substrate (lower) and monomers in THF solution (upper). PL decay profiles of TTPSB monomers in THF solution (b) and ensemble microbelts on Silica substrate (c).

Table S2. Photophysical parameters of TTPSB monomers in the dilute solution and microbelts.

Sample	λ_{abs} (nm)	λ_{em} (nm)	Φ^a	τ^b (ps)	k_r^c (ns^{-1})
**Monomer**	473	538<br>567<br>611	0.04	182±1.4	0.220
**microbelts**	470<br>543<br>566	583<br>631<br>666	0.15	425±2.3	0.353

[a]*Φ* of monomer solution in THF measured through a relative method by using Rhodamine 6G as a standard and *Φ* of microbelts measured through an absolute method by using an integration sphere. [b]Fluorescence lifetime. [c]Radiative decay rate calculated according to $k_r = \Phi/\tau$.

Stimulated emission of the pure crystal

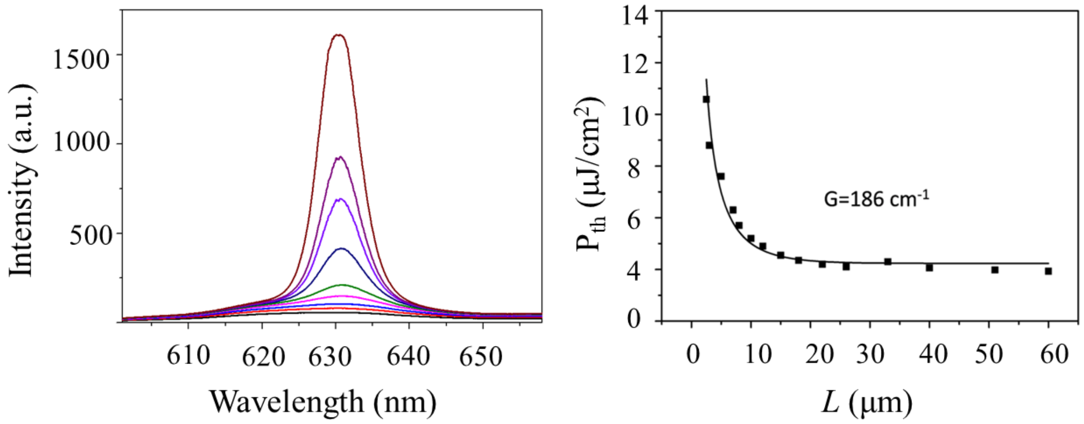

Figure S6. (a) Amplified spontaneous emission from individual TTPSB microbelts. The red box represents the gain region. (b) Threshold values as a function of TTPSB microbelt lengths, for example: 2.3 μm, 3 μm, 5 μm, 7 μm, 8 μm, 10 μm, 12 μm, 15 μm, 18 μm, 22 μm, 26 μm, 33 μm, 40 μm, 51 μm, and 60 μm.

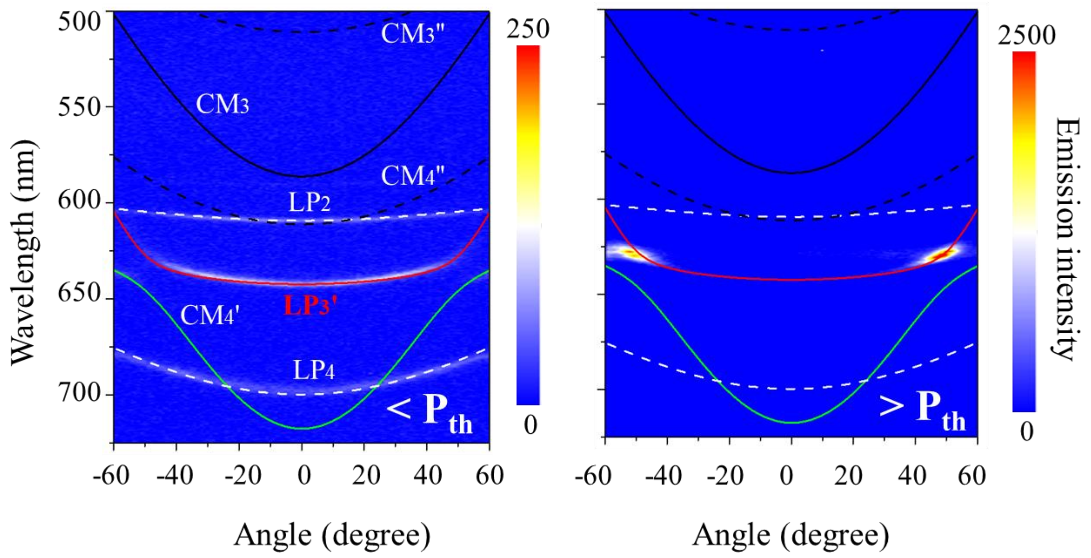

Figure S7. PL spectra below (left) and above (right) the threshold. LP_n denote the n-th lower polariton (LP) branch and CM_n denote the V-polarized uncoupled cavity mode (CM, black solid lines) respectively. CM_n" denote the H-polarized uncoupled cavity mode (CM", black dash lines), which derived from the theoretical simulation of the exciton polaritons. The H-polarized uncoupled cavity modes (CM_n") are invisible in the experimental results because they strongly couple with the excitons in experiments generating LP_n.

PL of samples in microcavity

Fig. S8a displays the emission spectra at the different pump fluence. In order to fully understand the change of the linewidth and blueshift below and above the threshold, we displayed the power-dependent k-space images (Fig. S8b-e) and the corresponding emission curves (Fig. S8f-i). At low pump fluence of P = 17.9 μJ/cm^2 (0.3 P_{th}), the LP dispersions of both LP_2 and LP_3 branches exhibit a broad and uniform emission distribution at all angles (Fig. S8b), which is agreement to the result of Figure 2a in the main text. This proves that the angle-resolved PL signal indeed originates from polariton emissions. The full width at half-maximum (FWHM) of this emission curve is fitted to be 2.90 nm (Fig. S8b). As the pump fluence increases, the LP_3 polariton intensity at θ = 40° exhibit rapid increase near two anticrossing points, whereas the LP_2 emission remains a little growth (Fig. S8f).

When the pump fluence reaches P = 65.6 μJ/cm^2 (1.1 P_{th}), the obvious new peak at 632.6 nm has been observed in the k-space images (Fig. S8c) and simultaneously a small portion of uncondensed residual of polaritons (existing at later moments of time) still displays the below-threshold dispersion, leading to two split modes (i.e., the condensed and uncondensed) coexist. The separation between the two modes is very clear at around k = 0, but not very distinguishable at larger k. In order to resolve the two modes in the single spectrum taken at the lasing k, we make the double-peak fitting for this emission curve (Fig. S8g). The condensed mode is fitted to be FWHM of about 1.75 nm and also exhibits a slight blueshift compared to spontaneous PL emission at 634 nm. Further increasing the pump fluence, the nonlinear increase of

the spectral intensities (Figure 3c in the main text) and spectral narrowing (Fig. S8d-e and Fig. S8h-i) confirm that the polariton lasing has occurred at two anticrossing points in the LP_3 branch.

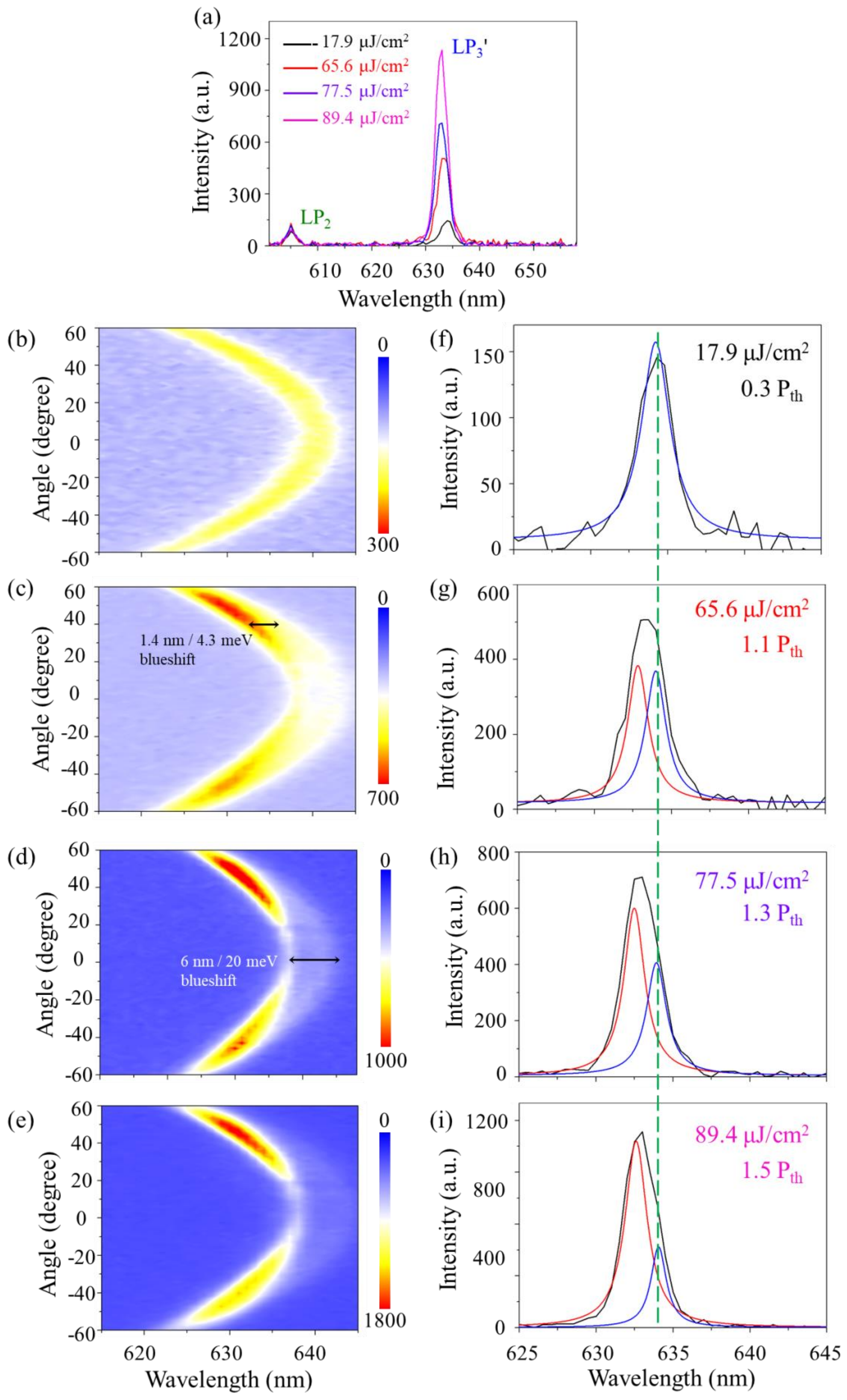

Figure S8. (a) PL intensities at θ = 40° as a function of pump fluence. (b-e) Angle-resolved PL spectrum below and above the threshold. (f-i) Fitted Lorentzian

line shapes for the calculation of FWHM.

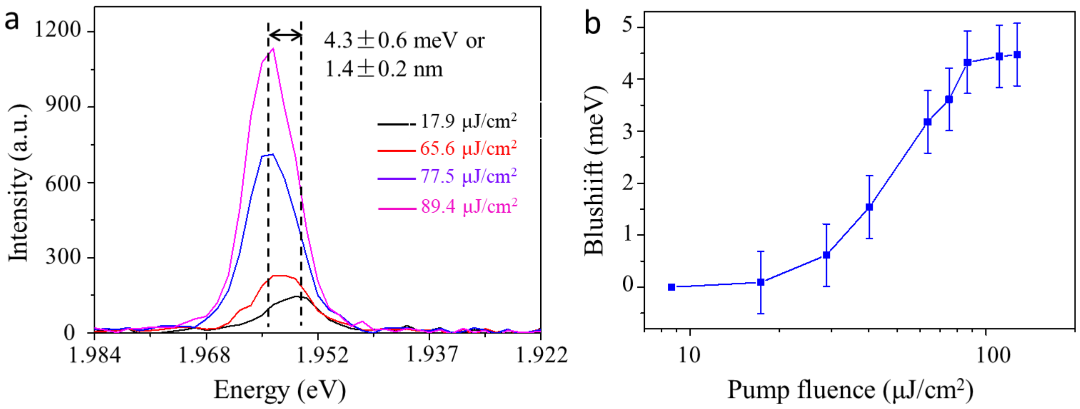

Figure S9. (a) The magnified figure of Figure S8a. (b) The spectral blueshift as a function of the pump fluence.

Based on the fact of two condensed and uncondensed modes coexist, we have to make the double-peak fitting for the emission curves above the threshold in order to well resolve the two modes in the single spectrum taken at the lasing k. Therefore, the correct blueshift value can be only obtained from the comparison between the spectrum above and below (in which only the uncondensed mode appears) the threshold, which gives the value of 4.3 ± 0.6 meV. The blueshift is clear, continuous and showing a definite value seen from the entire graph.

Table S3. Coupled Harmonic Oscillator Model Fitting Results for LP_1 to LP_4.

Coupling mode	LP1	LP2	LP3	LP4
**Rabi splitting (meV)**	140	520	590	592
**Detuning (eV)**	1.10	0.65	0.30	-0.1
$\lvert\alpha\rvert^2$	0.01	0.09	0.27	0.58
$\lvert\beta\rvert^2$	0.99	0.91	0.73	0.42

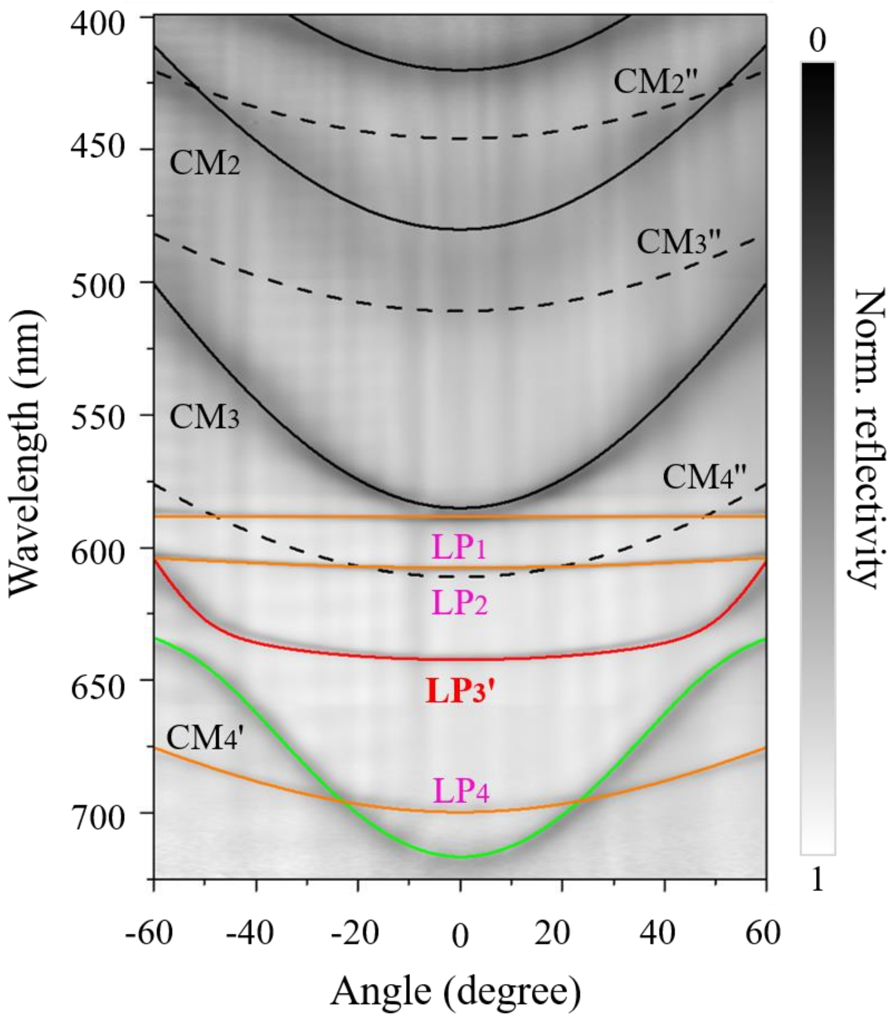

Figure S10. Angle-resolved reflectivity of the microcavity at room temperature. LP_n denote the n-th lower polariton (LP) branch and CM_n denote the V-polarized uncoupled cavity mode (CM, black solid lines) respectively. CM_n" denote the H-polarized uncoupled cavity mode (CM", black dash lines), which derived from the theoretical simulation of the exciton polaritons. The H-polarized uncoupled cavity modes (CM_n") are invisible in the experimental results because they strongly couple with the excitons in experiments generating LP_n.
